# Needs assessment in long-term care: expression of national principles for priority setting in service allocation

**DOI:** 10.1186/s12913-024-10889-1

**Published:** 2024-04-26

**Authors:** Ann Katrin Blø Pedersen, Marianne Sundlisæter Skinner, Maren Sogstad

**Affiliations:** Centre for Care Research, Department of Health Sciences in Gjøvik, Faculty of Medicine and Health Sciences, NTNU – Norwegian University of Science and Technology, Teknologivegen 22, 2815 Gjøvik, Norway

**Keywords:** Priority setting, Health policy, Needs assessment, Allocation, Older adults

## Abstract

**Background:**

Long-term care services for older adults are characterised by increasing needs and scarce resources. Political strategies have led to the reorganisation of long-term care services, with an increased focus on “ageing in place” and efficient use of resources. There is currently limited research on the processes by which resource allocation decisions are made by service allocators of long-term care services for older adults. The aim of this study is to explore how three political principles for priority setting in long-term care, resource, severity and benefit, are expressed in service allocation to older adults.

**Methods:**

This qualitative study uses data from semi-structured individual interviews, focus groups and observations of service allocators who assess needs and assign long-term care services to older adults in Norway. The data were supplemented with individual decision letters from the allocation office, granting or denying long-term care services. The data were analysed using reflexive thematic analysis.

**Results:**

The allocators drew on all three principles for priority setting when assessing older adults’ long-term care needs and allocating services. We found that the three principles pushed in different directions in the allocation process. We identified six themes related to service allocators’ expression of the principles: (1) lowest effective level of care as a criterion for service allocation (resource), (2) blanket allocation of low-cost care services (resource), (3) severity of medical and rehabilitation needs (severity), (4) severity of care needs (severity), (5) benefit of generous service allocation (benefit) and (6) benefit of avoiding services (benefit).

**Conclusions:**

The expressions of the three political principles for priority setting in long-term care allocation are in accordance with broader political trends and discourses regarding “ageing in place”, active ageing, an investment ideology, and prioritising those who are “worse off”. Increasing attention to the rehabilitation potential of older adults and expectations that they will take care of themselves increase the risk of not meeting frail older adults’ care needs. Additionally, difficulties in defining the severity of older adults’ complex needs lead to debates regarding “worse off” versus potentiality in future long-term care services allocation.

**Trial registration:**

Not applicable.

**Supplementary Information:**

The online version contains supplementary material available at 10.1186/s12913-024-10889-1.

## Background

The ageing population is increasing pressure on resources in long-term care services; thus, restructuring long-term care for older adults is on the political agenda in many countries [1, 2]. Consequently, there has been an increased focus on using care resources effectively and efficiently to improve the likelihood of desired health outcomes [3]. However, this focus has resulted in a range of challenges in long-term care service allocation, such as failure to meet citizens’ actual needs [4, 5], expanding budgets, rationing of care services, poorer quality of services and higher thresholds in service allocation [6]. To meet these challenges, priority setting and more effective care strategies, such as reorganisation of services and more efficient resource utilisation, have been foci of health and care services reforms in recent decades [6]. The aim of this study is to explore how national principles for priority setting are expressed in long-term care service allocation to older adults.

Universalism is a central feature of the Norwegian welfare model and gives all citizens the right to receive health and care services adapted to their needs. This means that municipalities are obliged to provide long-term care services that cover citizens’ needs for longer or shorter periods in situations that may threaten their welfare [6]. An increasing number of municipalities have in recent years adopted a purchaser-provider model for long-term care service allocation where those who decide on the scope of services given (allocators) are separate from those providing the services (providers). This means that needs assessment and allocation is carried out by municipal service allocators, who typically assess whether long-term care services should be provided, what service(s) should be offered, and, in the event of resource shortages, who should be prioritised [7].

For many years, in line with political aims in several countries, effort has been made to reduce the use of specialist care, decreasing the length of somatic hospital stays [8, 9]. In Norway, changes with reducing the length of hospital stays started in the late 1980s and have been expedited after the implementation of The Norwegian Coordination Reform in January 2012. This reform made the local municipalities responsible for parts of the citizens’ health care that were previously the responsibility of the hospitals [10]. Consequently, the local long-term care services are expected to deliver care to more service users and care recipients with more severe medical needs than previously [11]. These changes, in addition to the ageing population, increase demands for long-term care services and challenge the sustainability of universalism in the developed welfare state [12].

Norway’s National Insurance Scheme ensures free or highly subsidised health care and long-term care regardless of age [13]. Similar to most other Organization for Economic Cooperation and Development (OECD) countries, long-term care services are mainly publicly financed through general taxation and granted according to individual persons’ needs [14]. Citizens in need of long-term care can apply for several types of services and facilities, such as home care, sheltered housing, nursing homes, day-care facilities, personal assistance, and social alarms. Home care consists of various services, but in this study, we focused specifically on home nursing and home help (practical assistance). Sheltered housing is assisted housing that is allocated to older adults so that they can have easier-to-care-for housing and better access to facilities [15]. Some sheltered housing has staff on duty 24 h a day, while in others, residents must call for assistance when they need it [16]. Nursing homes are designed for older adults who require a high degree of medical care and assistance with activities of daily living. Short-term stays in nursing homes are provided to older adults who need health care at a higher level than home care for a shorter period [17], for example, as respite care, for observation to identify the level of care needed, or while waiting for a long-term placement. Additionally, short-term stays are used for preventive, treatment or rehabilitation purposes for older adults living at home [6, 18].

All Norwegian residents with illnesses or disabilities can apply for long-term care services in the municipality they live in [15]. The service allocators process applications, assess needs and make individual decisions regarding service delivery. A study including a sample of 804 service allocators from 261 municipalities indicates that the vast majority of municipal service allocators are nurses (> 80%), while the remaining are from other health professions or other (such as physiotherapists, occupational therapists or social workers) [19]. In effect, nearly all municipal service allocators are health professionals. Prior to service allocation, the service allocators obtain information about applicants by visiting their homes, sometimes with the next of kin present. At these home visits, the service allocators fill out assessment papers regarding the recipients’ needs and health condition and take notes about the recipients’ wishes, living situation, social situation, and family. If the applicants are already receiving long-term care services, the service allocators obtain the relevant information from service providers, relatives and the care recipient. In the assessment process, the service allocators can discuss with colleagues or long-term care staff before granting or refusing services. According to the Public Administration Act [20], applicants for long-term care services are entitled to a written reply with an individual decision provided by the municipality within 30 days of submitting the application for services. The individual decision letters contain the municipality’s arguments and reasons for granting or refusing long-term care services, as well as information about the right to appeal [20]. The applicants can send a new request for an assessment of their needs at any time. The content of the individual decision letters serves as the basis for the planning and provision of long-term care services in collaboration with the individuals who receive them [15].

**Fig. 1:**
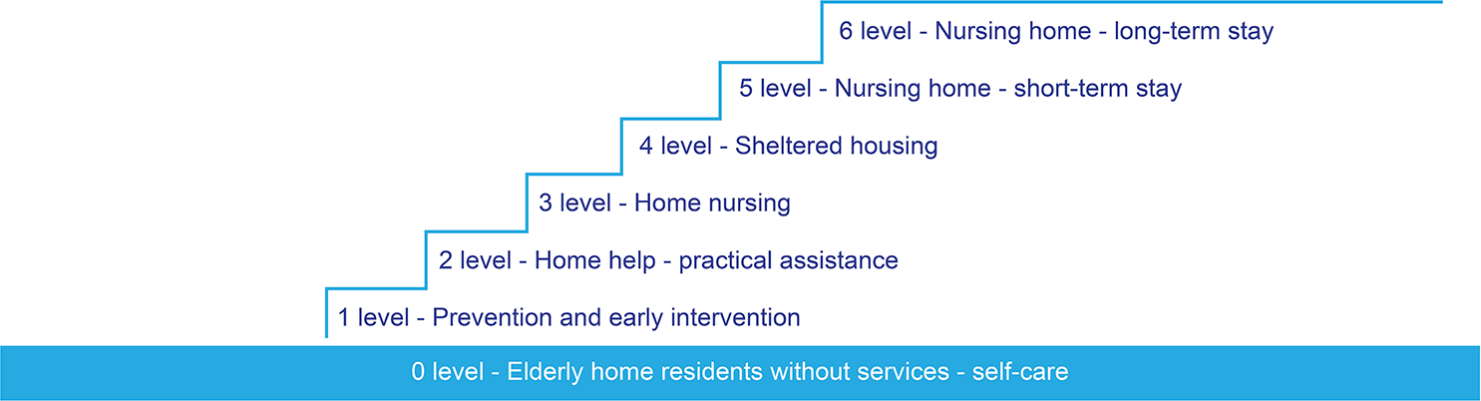
Norwegian levels of long-term care services

In Norway, the needs assessment and the allocation of long-term care services is often based on the principle of the ‘lowest effective level of care’, where services at lower levels (such as social alarms, level 1) should be tried before services at the higher levels (nursing home stays, levels 5 and 6) [17] (see Fig. 1). Home care services typically require fewer economic resources and serve less severe needs than institutional services [6] and therefore, in most cases, are offered before nursing home stays.

### Priority setting and resource allocation in long-term care

Priority setting in long-term care services is an underresearched area of study [17]. In the international scientific literature, there is a lack of clear definition of long-term care services [21]. This is because the organisation and responsibility for delivering these services differ across countries. Despite different systems, the core of the concept of long-term care is the need for support from a third party to manage activities of daily living (ADL) [22]. Furthermore, a primary challenge in long-term care service allocation is that citizens’ needs and/or demands often exceed the available resources, which leads to a need for priority setting [23]. Additionally, sustainable long-term care for older adults should also include the optimal distribution of resources [24]. To address the challenges and complexities in long-term care services, there is a need for a system for priority setting that is effective and just [7], that prevents exacerbation of illness and functional failure and ensures that persons in need of care get the right service at the right time. Health authorities are increasingly recognising the need for clearer principles for priority setting to ensure safe, efficient and equitable resource allocation [25].

Previous research on the topic has shown that constrained resources, health inequalities, and increased complexity in older adults’ health and care needs present challenges to priority setting in long-term care service allocation [24, 26, 27]. The consequences of these challenges can be marginalised care with ethical implications and less individualised and inclusive care [5, 28]. Despite service allocators’ desire to comprehensively assess older adults’ needs [29–31], limited resources influence the extent of needs assessed [30]. Earlier research has shown that in several cases, assessment and service allocation were more supply-led than needs-led [4, 30, 32, 33] and that service allocators were more loyal to the organisation and available resources than to the needs of service recipients [4, 30, 34]. A Norwegian study from 2018 showed that service allocators throughout the assessment process actively worked to fit older adults’ needs to services with low resources. As a consequence, certain older adults’ needs were obscured in the assessment process [34]. Older adults’ psychological and psychosocial needs are especially prone to remain unassessed [30].

Variations in long-term care assessment and allocation of services occur across regions, municipalities, and city districts both in Norway and internationally [19, 35–41]. Explanations of variations in service allocation are variations among organisations, municipalities’ economy, individual judgements, and the influence of resourceful and strong-willed relatives [17]. A study conducted by Syse et al. [19] showed that municipality size could explain some of the variation, as larger municipalities tended to allocate less practical assistance than smaller municipalities. Additionally, the individual service allocator to whom the care recipient was assigned was almost as important for the allocation of long-term care services as the municipality in which they lived. Some variations in service delivery are necessary and unavoidable due to differences in needs, preferences and clinical responses. Studies have shown that unwanted allocation variation occurs in services offered to younger and older service recipients [39] due to unequal standards in the assessment of care needs, with younger recipients’ needs being assessed relative to normal activities for those in the same age group [40, 41]. Variations in long-term care service assessment can be considerable in areas where no common priority-setting principles exist [37]. Principles for priority setting in long-term care service allocation can help establish a common foundation and reduce unwanted allocation variation [39].

Decisions regarding how health care needs should influence priority setting can draw on different theories of distribution, such as egalitarianism [42, 43], prioritarianism [44] and individualism [45] and on combinations of normative evaluations that underpin the principle of need [46]. A general normative principle of health care needs is that the larger the need for health care is, the greater the claim for such resources [46]. However, in the long-term care services, there are a myriad of variables independent of medical diagnoses that play a part in determining what care recipients’ care needs are. Contextual variables such as family networks and physical environment can be part of this. Therefore, the values, principles and criteria that should be the basis for priority setting in long-term care are subjects of debate [47]. Establishing commonly agreed-upon principles for priority setting is challenging due to values such as equality and fairness on the one hand, and resource constraints on the other [45]. Additionally, the absence of a common understanding of what long-term care services are and should be among policymakers, managers, service providers and service users as well as suboptimal measures of outcomes, impede formulation of public policy [21]. A study shows that employees in the Swedish long-term care services experienced national priority-setting principles in health care and nursing as useful [48]. Nevertheless, there is still a need to specify principles for long-term care services separately to primary and secondary care [49].

Recently, three principles for priority setting, resource, severity, and benefit, were selected as guiding principles in Norwegian long-term care services. These principles are the same as those in the Norwegian specialist health care, only with moderate changes [7]. However, there are major differences between the specialist and long-term care services, and therefore, it is not given that the three principles are equally adaptable in both sectors [50]. The long-term care service is a service that provides older adults early and long-term follow-up. The follow-up is often aimed at several diseases and disorders in addition to the consequences and challenges that the diseases cause (e.g., coping with everyday life, the functions of daily life and basic needs). The specialist health service, on the other hand, often focuses on one disease at a time, they complete the treatment and discharge the patient for further follow-up by the long-term care services [50]. Addressing these differences, in the principles for the long-term care services, the term “coping” have been incorporated in the severity and benefit principle [7]. This study explores how these three principles are expressed by municipal service allocators in the allocation of long-term care services to older adults.

## Methods

### Conceptual framework

Prioritisation means choosing something over or before something else, thus rejecting or postponing the less important or less urgent [51]. In an overall welfare state context, this is about decisions on distribution, redistribution and rationing of resources – in our context – long-term care resources [50]. Priority setting has been used to refer to the ways in which health and care measures are restricted due to economic constraints [45]. Economic constraints make the allocation of long-term care services more supply-led than needs-led [4, 30, 32, 33]. This contradicts the concept of universalism, which gives citizens the right to receive services based on needs [6]. In long-term care services, priority setting is about allocators choosing between different services and service users. Based on needs assessment, decisions are made to grant individual service users necessary services to enable them to live a good life with illness and loss of function. The objectives of quality of life, dignity and coping are central [7]. Needs assessment is often operationalised through a calculation of dependency [22] in order to provide help to maintain ADL and IADL (instrumental activities of daily living) functions [52]. However, in practice, the evaluation of dependency is challenging, as it is hard to measure [22]. This complicates priority setting in long-term care.

Explicit priority setting involves identifying principles that can guide allocation of scarce resources to ensure fair distribution [53]. Priority setting for Norwegian long-term care services is stated in the 2021 white paper on *“Benefit, resource and severity – Priority setting in the health and care services”* [7]. The white paper contains three guiding principles for priority setting: (1) the resource principle, (2) the severity principle and (3) the benefit principle. These three principles set the background for the research aim and provide an analytical framework for the findings of this study. The benefit principle and the severity principle in Norwegian long-term care include the ability to cope. Coping involves managing tasks and challenges throughout life, including mastering one’s own level of functioning and health-related aspects of life [7]. The 2021 white paper describes the three guiding principles as follows:

#### The resource principle

The fewer resources a measure requires, the higher its priority [7]. The use of resources refers to both the resources directly connected to the measure and the resources associated with the future use of services (e.g., preventive measures causing reduced use of services) [7].

#### The severity principle

The priority of a measure increases with the severity of the care recipients’ condition. The assessment of the recipients’ condition should be based on *“risk of death or loss of coping and/or function, the degree of loss of coping and/or physical or mental function, pain, physical or mental distress”* [7]. Additionally, the present situation, duration of the condition, and future loss of life years have an impact on the degree of severity. The more urgent the need to introduce the measure, the higher the degree of severity [7].

#### The benefit principle

The priority of a measure increases with the expected benefit of the measure. The assessment of expected benefits should be based on “*whether knowledge-based practice indicates that the measure can increase the patient’s life expectancy and/or quality of life by increasing the likelihood of survival, improvement, or reduced loss of coping and/or physical or mental function, reduction of pain, physical or psychological discomfort”* [7].

The white paper also states that the overall principle for priority setting requires the three principles to be assessed and weighted against one another. The higher the severity of a care recipient’s condition or the higher the benefit of a specific measure, the higher the acceptable use of resources. Allocating services in circumstances of low severity and low benefit can be justified only if the use of resources is low. The assessment of resource use will be decisive if two measures are deemed to be of equal benefit and severity [7].

### Design

The study design was qualitative, combining data from different sources: focus groups, individual interviews, observations, and administrative decision letters. The study is reported in accordance with the COREQ checklist (see Supplementary Material 2).

### Recruitment and participants

Service allocators were recruited from three municipalities. An invitation to participate in the study was sent to the heads of the health and care services in each of the three municipalities. To facilitate variation across settings, one small, one medium-sized and one large municipality were included in the project. Service allocators who assessed and allocated long-term care services to adults 65 years of age and above were informed about the study and asked to participate. Nine service allocators were recruited for individual interviews: two from the small, three from the medium-sized and four from the large municipality. To enhance “the information power” of the study, an additional three participants from the medium-sized municipality and one participant from the large municipality were recruited for focus groups. Information power refers to the richness of the data and potential of the sample to achieve the aim of the study [54]. All thirteen participants were women. Nine of the participants were nurses, two were physiotherapists, and two were occupational therapists. No relationship was established with any of the participants prior to the commencement of the study.

### Data collection

Data were collected in the period from August to November 2020.

#### The interviews

To explore the allocation of long-term care services to older adults, nine semi-structured individual face-to-face interviews were conducted. In addition, one focus group was conducted in the medium-sized and one in the large municipality, with five and six participants, respectively. In the small municipality, there were only two service allocators who administered long-term care services, so no focus group was conducted. All the interviews were conducted at the service allocators’ workplaces by the first author, a female PhD candidate with a master’s degree in health sciences. She introduced herself briefly at the start of each interview, declaring her assumptions, reasons for doing the research, and interests in the research topic. The individual interviews lasted between 45 min and one hour, and the group interviews lasted between 60 and 90 min. Both interview guides were developed for this project to explore service allocators’ experiences and dilemmas in the evaluation of older adults’ long-term care needs (see Supplementary Material 1). The individual interviews was made up of questions under five main topic headings: (1) Opening question: What are your experiences with needs assessment and allocation of services to older adults?; (2) Information and considerations in the allocation process for new care recipients; (3) Considerations of necessity and dilemmas in service allocation to older adults; (4) Changes in service allocation for existing care recipients; and (5) Collaboration in service allocation. The focus groups focused on clarifying dilemmas that emerged as salient in the individual interviews and observations.

#### Observation and informal conversations

The study involved observations of weekly allocation meetings, carried out by the first author. In these meetings, the allocation of services for individual service users was discussed, particularly which older adults should receive nursing home stays. The participants in these meetings were either service allocators only or service allocators *and* long-term care staff, such as nurses, physiotherapists, occupational therapists, and managers of nursing homes. The first author observed 14 of these meetings across the municipalities. The meetings lasted 60–90 min. An observation guide was used; the observations focused on what the participants in the meetings emphasised when discussing the allocation of long-term care services to older adults. To enhance comprehension of the observations, clarifying questions were posed to the 13 service allocators both prior to and following the observations. Additionally, 140 h of observation of service allocators’ daily practice were conducted. Field notes were taken during the interviews and observations, and any data that could identify care recipients were left out of the notes.

#### Individual decision letters

Service allocators from each municipality were asked to provide anonymised individual decision letters. We received 64 such letters in total, with 38 from the large municipality (of which approximately 20 were related to one older adult care recipient following his trajectory of care), 19 from the medium-sized municipality, and seven from the small municipality. All administrative decision letters were included in the study. Throughout the results section quotes will be identified by M: Municipality; G: Group interview; I: Informant; DS: Denied service; GS: Granted service.

##### Ethical approval and consent to participate

Ethical approval was granted by the Regional Committees for Medical and Health Research Ethics, REK North (2020/111,946), and Norwegian Centre for Research Data (NSD; reference number 693,007). The collected confidential documents regarding individual decisions were anonymised and treated as confidential, in accordance with the REK North requirements. The service allocators participating in the study were assured of confidentiality and anonymity, and written informed consent was obtained from all participants prior to participate in the study. The service allocators were informed about the rationale behind the study and that they could withdraw from the study at any time. All individual decision letters were anonymised before we received them. The study was performed in line with ethical standards set forth in the 1964 Declaration of Helsinki and its later amendments.

### Coding and data analysis

We conducted a reflexive thematic analysis inspired by Braun and Clarke [55], including six analytic steps in which a latent approach was taken. This analytical process was a collaboration among the three authors. The first analytic step was becoming familiar with the data. All the interviews were conducted and audio-recorded by the first author and transcribed verbatim by the first author and a research assistant. All the interviews, field notes and administrative decision letters were closely read, and initial ideas were written down. The second step was generating initial inductive codes. The initial coding resulted in approximately 700 codes. For systematic organisation, review, and analyses, NVivo 20 software was used. Step three was searching for themes. Here, we used a deductive approach whereby the three principles of priority setting (resource, severity, and benefit) were used as an analytical framework to sort the initial 700 codes into themes. In the fourth step, we reviewed the themes, and in step five, we defined and named the themes, resulting in six themes. Step six was writing the paper. The analysis involved movement back and forth among the six steps. The first author conducted the first two steps of the analysis, whereas all three researchers were involved in steps three to six and critically reviewed the themes and reread the codes, ensuring a joint understanding and interpretation of the results.

## Results

The findings reveal the need to prioritise limited long-term care resources. Six themes were derived from the analysis of the interviews, the field notes, and the individual decision letters, with two themes related to each principle (Table 1).

**Table 1:** Themes

Principles	Themes	Citation
Resource	Lowest effective level of care as a criterion for service allocation	*“Home care services must be tried before you meet the criteria for short-term stays”* (M3, I7).
	Blanket allocation of low-cost care services	*“Social alarms* […] *are a low-threshold service, so everyone who asks for it gets it” (M2, I5).*
Severity	Severity of medical and rehabilitation needs	*“According to the allocation criteria for long-term stay, there must be a somatic health failure and/or a dementia diagnosis that requires extensive medical treatment”* (M2, DS). *“You were hospitalised due to a femoral neck fracture. After the hospital stay, you had a longer short-term stay at the nursing home for rehabilitation and medical treatment. You have gradually recovered physically, but still have varying health and motivation for exercise. The rehabilitation team will provide services for a period of training after you have returned home from the nursing home”* (M3, GS).
	Severity of care needs	*“It is more difficult to motivate patients to receive services at home if the unease and anxiety is so great that we are not able to calm them”* (M1, I1). *“As a result of reduced short-term memory and reduced cognitive function, there is a need for increased assistance from home nursing to ensure personal hygiene and that you eat”* (M3, GS).
Benefit	Benefit of generous service allocation	*“Many times, we want to secure the elderly who are discharged from hospital by allocating four home nursing visits a day, for example, at the start. Because it is a bit uncertain in the beginning.* […] *And then the home nursing staff may think that we have allocated far too much. And it may well be that they are right sometimes but wrong other times. And especially in transitions, I think we should be generous in the transition, and subsequently reduce* [the level of services] *instead”* (M3, I7). *“We have services such as reablement which we really should invest even more in.* […] *Yes, for example, a reablement team that comes in and does exercises regularly for a certain period so that the older person may be able to go to the shops on their own”* (M3, I8).
	Benefit of avoiding services	*“We have things that can be ordered so you can get your socks on. Stocking pullers and things like that. And it is important to focus on coping and what kind of resources they* [the recipients] *have”* (M1, I2). *“Because if we go in with too much help, then… we shouldn’t deprive them of their autonomy over everyday life either. We are not going to take over functions they can handle themselves. So, it is very important to find out: ‘What can you do yourself?’ So that they can cope and experience that”* (M1, I3).

**Abbreviations**: M: Municipality; G: Group interview; I: Informant; DS: Denied service; GS: Granted service

**Fig. 2:**
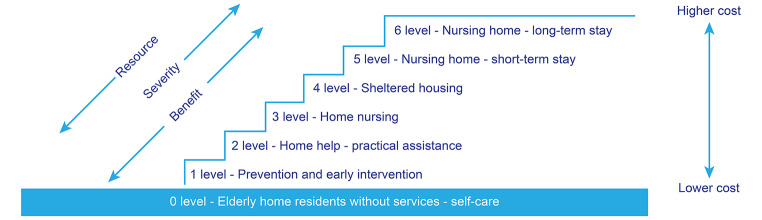
The influence of the three principles of priority setting on service allocation

Overall, the analysis shows that the service allocators’ practice reflects the three principles of priority setting. The principles influence the service allocation process in different directions in the needs assessment and allocation of services to older adults (see Fig. 2). The resource principle is expressed through the allocation of low-cost services, thus pushing the allocation of services to a lower level of care. In cases of unsafe and undignified situations for older service recipients, the severity principle pushed the allocation of services to a higher level. In cases where services would potentially lead to the recovery of function, the benefit principle was expressed, pushing the allocation of services to a higher level. In contrast, the benefit principle was also expressed when it was perceived that older adults would profit from *not* receiving (more) services. Here, the logic was that receiving fewer services or services at lower levels of care would benefit care recipients’ independence and their ability to cope and maintain everyday function. Thus, the benefit principle could push allocation of services to either a higher or lower level of care.

### The resource principle

Our analysis shows that the resource principle was expressed through the ‘lowest effective level of care’ as the leading criterion for service allocation and through extensive allocation of low-cost long-term care services to older adults.

### The lowest effective level of care as a criterion for service allocation

The service allocators explained that before older adults qualified for short-term or long-term stays in a nursing home, services in the home would have to be tried. This practice is illustrated by the reasoning in an individual decision letter that rejected an application for a long-term placement in a nursing home: *“Here, special emphasis is put on whether all measures regarding practical help and necessary health care have been attempted to be provided at home”* (M1, DS). Services would be attempted to be provided in the home through up to five or six visits a day by home care services providers before a stay in a nursing home would be considered. Alternatively, when care recipients needed observation and evaluation of their needs throughout the day and night, short- or long-term stays in nursing homes were allocated. Although the service allocators explained that the “lowest effective level of care” alone was not sufficient in the assessments, they described a main act of allocations of low-cost services and expressed that the “lowest effective level of care” was a leading criterion for service allocation to older adults.

### Blanket allocation of low-cost care services

The services allocators’ expressions of their practices revealed extensive allocation of low-cost services, such as practical assistance and social alarms. Applications for these kinds of services were hardly ever rejected. One service allocator explained that low-cost services were sometimes provided even in cases when care recipients did not need them: *“So what I’m saying is that we may in fact have given practical assistance to older adults who strictly do not need it”* (M1, I2). Several of the service allocators emphasised that in future assessments, they would have to be stricter when allocating home help, such as practical assistance, due to resource and capacity shortages. The service allocators explained that all applicants for the social alarm, which is a service at the lowest level of care, were granted the service without any needs assessment: *“Then there are social alarms, which is low threshold offer so everyone who asks for it gets it”* (M2, I5). They explained that the social alarm reduced unnecessary home nursing visits, as service recipients could use the alarm when they needed assistance. However, the service allocators emphasised that the social alarm could provide older care recipients with a false sense of security, as they could gradually lose their understanding of how to use the alarm due to an increasing risk of cognitive impairment. This, it was explained, could lead to unsafe situations in which older adults’ needs were not discovered and thus not met. Hence, when service allocators extensively allocated low-cost services, this could result in uncovered needs or the allocation of services that were not actually needed.

### The severity principle

Allocation based on the severity of older adults’ situation was expressed through medical and rehabilitation needs in response to reduced physical functional level where ability to cope was essential. Additionally, the severity principle was expressed through older adults’ care needs. Severity assessments of care needs were based on evaluations of basic needs in activities of daily living. Such needs were described by the service allocators as hygiene, nutrition, and feeling secure. In addition, the service allocators emphasised dignity and whether older adults could take care of themselves.

### Severity of medical and rehabilitation needs

The situations that were described as highly severe and unsafe were older adults being unable to cope with their medical condition caused by frailty, illness or cognitive impairment. In particular, cognitive impairment in combination with somatic health failures (e.g., diabetes or bone fractures) affected older adults’ functional level (measured as ADL and IADL) and ability to cope. In severe situations where the care recipients had needs that required extensive medical treatment over time, the service allocators allocated long-term nursing home stays. In situations where short-term medical follow-up and rehabilitation were needed, short-term nursing home stays were allocated. When it was considered safe for the care recipients to be sent home, they were provided with home care services to cover their medical and rehabilitation needs. The service allocators explained that decisions regarding medical needs and safety were made in collaboration with doctors: *“In the nursing home, it is often the doctors who, sort of… point out that it is medically unjustifiable that the older adults are not in an institution”* (M3, I7).

The service allocators also considered whether a short-term stay at a nursing home was sufficient or if the care recipient’s functional level was so low that they needed a long-term stay at the nursing home. In some cases, older adults had maximised their rehabilitation potential in the short-term unit at the nursing home but still had a reduced ability to cope with their condition. Then, the service allocators assessed the older adults’ situation as too severe to send them back home to be cared for by the home care services: *“The patients who are in a short-term rehabilitation unit who don’t get worse, but who don’t get better either, need* […] *a long-term stay* [in a nursing home]*”* (M1, I1). The allocators explained that these assessments of long-term stays were frequently performed in collaboration with nursing home staff and could be based on older adults’ ability to cope with their physical functional level, medical needs and need for care.

### Severity of care needs

The severity of older adults’ need for care was based on risks associated with frailty, falling and cognitive impairment, such as memory loss and disorientation, resulting in the need for supervision and/or assistance. These conditions could cause severe situations, such as care recipients not being able to cover their basic needs such as eating, toileting or turning off the cooker. The allocators thought it was challenging to determine the extent to which older adults could take care of themselves in their own home, especially in cases of psychological or cognitive impairments. One service allocator pointed out that older adults’ cognitive function could affect the severity of their situation: “*Many things can work well at home as long as* [the care recipient] *is clear and oriented. They may have an incredibly large functional decline, and it can still work because they understand that they need to call for assistance”* (M1, I4). The service allocators explained that the level of care for a given care recipient was often discussed with home nursing staff and in some cases affected the allocation outcome. One service allocator stated: *“Sometimes they* [the home nurses] *say, ‘There is no point; it is only going to end in a fall, anxiety and uneasiness and little coping’. In addition, then we take that with us, and there is no point in trying a lower level of care”* (M1, GI, I1). It was also clear that service user preferences or differences in older adults’ situations could influence service allocation. Examples of this could be wishes expressed by the care recipient or the family, variations in the layout of the home and ability to adapt the environment to their needs, or the (limited) availability of informal caregivers: *“You can have two adults who have a relatively similar or identical condition at admission* [to the nursing home] *but who have completely different circumstances at home, which means that they receive completely different* [services]*”* (M3, I9).

The question of dignity also affected the evaluation of severity and was linked to the care recipient not being able to go to the toilet, being in a palliative phase, being lonely or sad, or having anxiety. The service allocators reported that insufficient numbers of nursing home places caused undignified situations for older individuals. The service allocators described how in some cases, they were faced with resistance and questions from both their own leaders and managers and doctors at the nursing homes when they allocated nursing home stays: *“We get a lot of criticism for allocating places at the nursing home too quickly, which we disagree with. But in these cases* [our argument] *is about safety and dignity”* (M3, GI, I10). The service allocators reported that it was more challenging to assess the severity of care needs than the severity of needs for medical care and rehabilitation, especially in allocations of nursing home stays. All these factors affected the severity assessment in the service allocation, and in cases of scarce resources, older adults who were considered “worse off” were allocated higher levels of services.

### The benefit principle

The benefit of services was expressed by the service allocators in two contradictory ways: generosity in service allocation and avoidance of service allocation.

#### The benefit of generous service allocation

The service allocators described the benefit of generosity for older adults in cases where the ability to rehabilitate and increase coping was likely. Generosity was especially emphasised during service transitions from hospital or nursing home to home based on the argument that extended care delivery would increase the likelihood of rehabilitating older adults’ conditions and postpone the later need for more extensive services. The allocation of services that cause a reduced need for services in the future is also in accordance with the resource principle. The service allocators noted that being generous and actively engaging in older adults’ rehabilitation before the transition from nursing homes to home could help older adults regain function more quickly and thus return to their home more quickly. A service allocator explained: *“Work even more actively in relation to those who are in a short-term stay at the nursing home to get them functional and make the short-term stay as short as possible”* (M3, I7). Although the service allocators often remarked that they tried to make nursing home stays short and tried to avoid allocation of nursing home stays for as long as possible, the findings show that when they expected that the older adults could benefit from extended or repeated short-term stays, the stays were allocated. The care recipients’ benefit from such services was explained as a better ability to rehabilitate and cope with everyday life and to stay at home for as long as possible.

The service allocators explained that in many cases, it was beneficial for the older adults to stay at home for as long as possible; therefore, they allocated services with the intention of preventing and postponing the transition from home to the nursing home. In some cases, sheltered housing was allocated to reduce the care recipients’ need for extensive care services and chance of requiring a long-term nursing home stay. Additionally, extended rehabilitation at home was explained as more beneficial for some older adults: *“Because we see that when older adults are at home, they are not as passive as when they stay in the nursing home.* […] *So, we see that when we can get* [them] *straight home again, they recover faster”* (M3, I7). When the service allocators assessed the level of care and the amount of care provided, they emphasised the recipients’ ability to recover and to prevent extensive need for services, as this was seen as beneficial to them and their health. The higher the service allocators’ expectation of the recipients’ possibility of recovering or being rehabilitated, the more generous the allocation of services.

### Benefit of avoiding services

The benefit of avoiding service allocation was identified when the allocators believed the older adults would be able to cope and better maintain their functional level without services. A service allocator explained that the more older adults’ benefits increased, the fewer services they were allocated: *“*[We] *think of it a bit like a gift that keeps on giving; solving it at the lowest possible level of care gives so much coping and quality of life”* (M1, I4). The service allocators said that they look for the care recipients’ inherent resources to obtain information about what they can master themselves and do not need help with so that they can control and cope with everyday life to the greatest extent possible. In some cases, the service allocators stated that they gave advice and guidance to older adults on how to cope with everyday activities, resulting in the avoidance of service allocation.

The service allocators’ expectations of older adults’ ability to take care of themselves are also reflected in the individual decision letters on granted home nursing care and reduced practical assistance: *“*[You will] *receive increased assistance until you master this again”* (M2, GS), *“it is important that you perform the daily tasks you manage yourself”* (M1, GS) and *“Everyday activities are important to maintain good health and independence”* (M3, RS). The service allocators also explained that they expected older adults to think preventively themselves and be proactive and adaptive so that they could cope with their everyday life in the future. Staying in shape with exercise, adapting their home or moving to a one-plan apartment, and seeking out activities in the municipality were examples the service allocators gave of preventive measures that older adults could both benefit from and initiate themselves without receiving services.

## Discussion

This study provides insights into how service allocators reason and justify their decisions and priorities when allocating long-term care services to older adults. Our analysis focused on how the three guiding principles of resource, severity and benefit were expressed by service allocators. The findings show a complex process in which many aspects are considered. The expressions of the three principles of priority setting push in different directions in the needs assessment and allocation of services to older adults. The findings show that the principles are expressed in accordance with current political trends and discourses: “ageing in place”, active ageing, an investment ideology (focusing on coping and rehabilitation) and prioritising the “worse off”. These political trends and the results of this study are discussed below.

### “Ageing in place” and active ageing: A matter of coping at home

Overall, this study shows that the threshold for allocating nursing home stays is high and that service allocators emphasise older adults’ ability to cope and live at home as long as possible. That Norway has a comparatively well-developed home care service with a range of specialised services and teams makes this possible [56]. To enable older adults to cope in their homes, service allocators extensively allocate low-cost services, such as social alarms and practical assistance. Our findings are in accordance with the policy of “ageing in place” that intends to meet older adults’ needs while enabling them to remain independent and simultaneously reducing costs [6, 57]. The proportion of older adults over the age of 67 who receive long-term care services in Norway has gradually decreased since the early 1990s. Particularly regarding nursing home services, the threshold for being allocated a stay has been raised considerably [58]. Our findings also indicate that when service allocators evaluate older care recipients’ condition or situation as too severe, unsafe or undignified for them to remain at home, they tend to allocate nursing home stays. However, it is clear that service allocators are sometimes unable to allocate nursing home stays due to a shortage of places. Thus, our results show that an extensive focus on coping strategies to enable “ageing in place” and scarce nursing home places in long-term care can lead to unsafe situations and unmet care needs.

The service allocators’ focus on coping strategies when allocating services to older adults also relate to international policy perspectives and discourses of ‘autonomous ageing’, ‘healthy ageing’ and ‘active ageing’ [59, 60]. The active ageing perspective developed as a protest against earlier perspectives of old age as negative, passive, and associated with morbidity and withdrawal from society [60]. Active ageing replaces these earlier perspectives with perspectives of ageing as positive, active, and healthy [60]. Our results show that service allocators commonly emphasise services such as reablement and short-term nursing home stays to activate and develop coping strategies for older adults. The allocation of services is often time-limited, and services are withdrawn when recipients can again master the necessary tasks themselves. In recent years, there has been increased implementation of reablement services in Norway [61]. Reablement can be defined as *“a person-centred, holistic approach that aims to enhance an individual’s physical and/or other functioning, to increase or maintain their independence in meaningful activities of daily living at their place of residence and to reduce their need for long-term services”* [61]. This definition shows, as found in our study, both the intention of activating older adults with coping strategies and the preventive purpose of the allocation of reablement services.

Although the perspective of active ageing has positive connotations, it has been criticised for putting unreasonable demands on older people to focus on health, social integration, and self-control [60]. Our study suggests that service allocators expect citizens to plan, cope and take care of themselves in their old age by being active, social, and adapting their homes. This focus on activating older adults through self-control and self-care in long-term care is not a new concept [62]. In 1989, Martinsen [62] emphasised that the caregivers of older adults must strengthen the care recipients’ ability to help themselves. However, when older adults’ independence, coping and ability to think preventively are emphasised in allocations, the responsibility for care is transferred to the older adults themselves, which increases the risk of not meeting frail older adults’ care needs [63]. Through compensatory care services such as practical assistance or help with hygiene, it is increasingly expected, as was also found in this study, that older adults should be motivated to manage and take care of themselves [63]. This enhanced focus on coping strategies, prevention and activation in long-term care service allocation to older adults is at least in part a result of political approaches intended to reduce cost and to establish sustainable long-term care services [23]. These strategies are discussed below.

### An investment ideology: coping and rehabilitation

Our findings suggest that service allocators emphasise older adults’ coping and rehabilitation potential when assessing recipients’ needs and service allocation. There is a willingness to grant services that require more resources, such as reablement services, if older adults have rehabilitation potential. This is logical from an investment perspective [23]. The investment perspective is exemplified through more attention to prevention, rehabilitation, activating care, and coping strategies [64]. Notably, greater emphasis is placed on allocations of short-term nursing home stays that are used for preventive, treatment or rehabilitation purposes aimed at enabling older adults to live at home [6] based on the argument that these measures empower the service users [65]. If reablement leads to older adults becoming more self-supporting, then municipalities can save resources by introducing reablement programs and additionally providing services to older adults with chronic long-term care needs [59]. However, it is also relevant to question whether this investment perspective, as is also indicated in this study, will lead to lower priority for user groups that do not benefit in the form of increased coping and self-support [64]. This could result in older adults with chronic needs being given lower priority than older adults with rehabilitation potential [23].

Another possible consequence is that older adults as a group are given lower priority than younger service users, who have more to gain in terms of ability to cope, being self-supporting [66] and being more likely to be motivated to become independent [65]. It is questionable whether reablement activities appeal to older recipients to the same degree as younger recipients, as most older adults cannot return to their life as it was before they became frail [60]. Another issue is that such activities may be at odds with older care recipients’ idea of a dignified and meaningful everyday life leading to frailty and death [67]. Thus, the appropriateness of ascribing such importance to the reablement potential of older adults is questionable, as is whether they are beneficiaries or victims of such a policy [60]. Our results show that service allocators rarely consult older adults or their families directly about their wishes. This blocks user participation and is likely to have a negative impact on quality and safety in service allocation [68]. To avoid victimising older care recipients, implementing a goal-directed reablement can potentially contribute to control over goals and activities and empower older adults in the reablement process [69]. However, compared to other countries [70, 71], the Norwegian long-term care model leans heavily on health and nursing as foundation of care to older adults and is not organised as a part of social service delivery [72]. It has been highlighted that nursing-based care services fail to address care recipients’ social care needs [73]. A lack of set standards leaves it up to the service provider to determine whether social care needs are met [72]. A recent systematic review found that both quality of life and cost-effectiveness were positive effects of social work interventions for older adults [74]. Thus, more diverse, multi-professional allocation teams and long-term care workforce is needed [72] to adequately meet older adults’ care needs and enable them to cope with their conditions and lives.

Our findings show that service allocators find it easier to assess medical and rehabilitation needs than care needs. It appears that there are more conflicts between allocators and other staff in municipal health and care services regarding the allocation of services covering *care* needs than the allocation of services covering medical and rehabilitation needs. This may be caused by the above-mentioned lack of integrated long-term care, causing conflicts between the nursing-based care service and services concerning dignity and quality of life. For decades, there have been concerns that the search for more effective and clearer measurable outcomes for service allocation turns attention to nursing tasks that fall closest to ‘cure’ (medical and rehabilitative) tasks, as they are easier to quantify than ‘care’ tasks. Cure tasks can be defined as *“episodic, sparked by the appearance of symptoms”*, and care tasks can be defined as *“continuous, often evoked by a chronic condition”* [75]. For many people, ageing is accompanied by chronic conditions, frailty and reduced functional capacity that the trend of active ageing and the investment perspective tend to ignore. In 1988, the gerontologist Moody [76] criticised the theory of activity as the solution for old-age problems, as it contributes to an illusion of old age as a disease that can be cured. This concern is still relevant when attention to investment strategies focused on coping and improvement potential, as was found in this study, serves as the basis for service allocation for older adults.

High focus on coping and improvement potential rejects biologically conditioned impairments moving towards frailty and death and gives rise to the perspective that old age can be cured [60]. Older adults receive long-term care services because of factors such as frailty, diagnoses or diseases, or combinations of these factors related to old age that result in a reduced functional level. Our study shows that service allocators allocate short-term nursing home stays and time-limited reablement services and emphasise that older adults should regain their function or potentially prevent reduced functional levels by engaging in reablement activities. When older adults show “symptoms” of old age by a reduced functional level, service allocators look for improvement potential as the basis for the allocation of services.

### Prioritisation of the “worse off”

The service allocators’ expressions of severity in service allocation in this study show that the evaluations of older adults’ needs and, in the event of resource shortages, of who needs services the most (who is “worse off”) are complicated and based on professional evaluations of recipients’ basic needs and safety. Basic needs that qualify for service allocation are hygiene, nutrition, and feeling safe. Safety is related to risks in connection with cognitive, physical, and medical conditions. Our findings show that the expression of the severity principle is especially prominent in cases where older adults are competing for nursing home stays due to a shortage of places in the municipality. In these cases, the service allocators decide which care recipient is “worse off” and needs the nursing home stay the most. Barra et al. [25] argue that assessments based on severity are controversial and ethically ambiguous and that there will always be disagreement about when and to what extent a condition is seen as severe.

Our study shows that assessing the severity of older adults’ needs is a highly complex process in which several characteristics affect the assessment, showing that the principle of “the larger need, the greater claim for services” [46] does not always apply. Assessments of frailty, diagnoses and diseases must be combined with evaluations of individual coping potential where factors such as home environment and living arrangements are relevant. This affects the evaluation of the severity of older adults’ situations. These complex characteristics of older adults’ needs make it difficult to set parameters regarding which older adults are seen as having a larger need and thus being “worse off”.

### Strengths and limitations

This study sheds light on how service allocators express and negotiate the three national priority principles, resource, severity and benefit, in long-term care service allocation to older adults. A qualitative approach was used to capture service allocators’ experiences and dilemmas in the evaluation of older adults’ long-term care needs. A strength of the study is that it uses data from several sources, interviews, observations, and individual decision letters, which is a confirming strategy that contributes to increasing its validity. The trustworthiness of this study is strengthened by the use of interview guides in the data collection phase [77] and by the involvement of all three authors in the analysis, ensuring the dependability and consistency of the findings [78]. The first author conducted the data collection and then critically discussed the results with the research team, which ensured a good balance of closeness and analytical distance to the data [79].

The informants mainly represented the nursing profession, and they were all women. This study might have benefited from having male participants and a higher representation of other professions. However, since the service is staffed mainly by women, and nursing is the most common profession among Norwegian service allocators (> 80% are nurses) [19], we consider the sample to be representative of the setting under study. However, the transferability of this study to other contexts might be restricted by variations in the service settings of long-term care in different countries. Nonetheless, since other welfare states are facing challenges similar to those experienced in Norway and the answers to these challenges are the same across different countries (active ageing, reablement, and coping), we believe that the study and its results have relevance outside Norway, irrespective of service organisation.

## Conclusions

This study reveals that service allocators’ expressions of Norway’s national priority-setting principles are in accordance with broader political trends and discourses, such as “ageing in place”, active ageing, an investment ideology, and prioritisation of the “worse off”. Some of the trends and results examined in this study tend to give more attention to services with improvement potential, which are easier to quantify than care services aimed at chronic needs [75]. In addition, expectations that frail older adults should take responsibility for their own situation to a greater extent entail a transfer of the responsibility of care to older adults themselves. Both the increasing attention to care recipients’ rehabilitation potential and expectations that older adults will take care of themselves increase the risk of allocations not meeting frail older adults’ care needs. Bringing in more diverse, multi-professional perspectives into the allocation teams and/or processes can be one way of reducing this risk; involving care recipients and their families in decisions is another.

The high focus on activity through coping and improvement potential in service allocation to older adults rejects their biologically conditioned impairments towards frailty and death. With extensive focus on improvement potential in the context of shortages of nursing home places, the severity aspect is at risk of being underestimated. Furthermore, the complexity of older adults’ needs makes severity difficult to define. Nonetheless, this raises professional and ethical debates regarding the prioritisation of those with rehabilitation potential over those who are “worse off” in future long-term care service assessments and allocations.

### Electronic supplementary material

Below is the link to the electronic supplementary material.

Supplementary Material 1

Supplementary Material 2

## Data Availability

The data will not be shared. Ethical approval for the study requires that the administrative decisions and transcriptions of the interviews be kept in locked files, accessible only by the authors. The data is available from the corresponding author on reasonable request.

## Abbreviations

ADL: Activities of daily living
IADL: Instrumental activities of daily living
M: Municipality
G: Group interview
I: Informant
DS: Denied service
GS: Granted service
RS: Reduced service
