# Supplementary material for: Needs assessment in long-term care: expression of national principles for priority setting in service allocation
Source: BMC Health Serv Res. 2024 Apr 26;24:530. doi: 10.1186/s12913-024-10889-1 (PMC11046954; doi:10.1186/s12913-024-10889-1)
Supplement: Supplementary file 1 — Supplementary Material 1 [file 12913_2024_10889_MOESM1_ESM.docx]

# Individual interview guide

**1. Experiences with service allocation to older adults**

What are your experiences with needs assessment and allocation of services to older adults?

Can you describe the service allocation process?

How do you feel about assessing and making decisions about service allocation for older adults?

**2. Information and considerations in the allocation process for new care recipients**

How do you collect information about care recipients when making the initial allocation decision?

How do you assess the scope of services allocated?

Which frameworks affecting the allocation process, do you have to relate to?

Do you experience a need for rationing when allocating services?

In your experience, in what ways is professional judgment used in needs assessment and service priority setting?

In your experience, are there any other factors that influence first-time decisions?

**3. Considerations of necessity and dilemmas in service allocation to older adults**

How do you assess the scope of necessary and appropriate health and care services for older adults?

What do you emphasise the most when assessing necessary and safe health and care services for older adults?

What do you emphasise in the written decision letter when justifying necessary and safe health and care services for older adults?

Do you experience ethical and professional dilemmas when assigning services to older adults? Please describe.

**4. Changes in service allocation for existing care recipients**

How is the allocation of services adjusted when there are changes in the needs of existing care recipients?

Which factors contribute to adjustments in service allocation to older adults?

Are there any challenges when service allocation is to be changed? Please describe.

**5. Collaboration in service allocation**

Does the municipal management set any boundaries for your evaluations and decisions about allocating services to older adults?

Are there other healthcare professionals who have an impact on your assessments and decisions about service allocation to older adults?

**6. Summary**

Is there anything I haven't asked about that you think is important when it comes to allocating services to older adults?

# Group interview guide

**1)** How does the lowest effective level of care affect your needs assessment and service allocation to older adults?

**2)** How do you think cuts in nursing home places affect priority setting in service allocation?

**3)** What are your views on dignity? Do you have examples of conditions or situations that are undignified?

**4)** Do you think the services allocation process is fair?

**5)** How is prevention emphasised in needs assessment and allocation of services to older adults?

**6)** In what ways is user participation emphasised in the assessment and allocation of services to older adults?
