# Supplementary material for: Needs assessment in long-term care: expression of national principles for priority setting in service allocation
Source: BMC Health Serv Res. 2024 Apr 26;24:530. doi: 10.1186/s12913-024-10889-1 (PMC11046954; doi:10.1186/s12913-024-10889-1)
Supplement: Supplementary file 2 — Supplementary Material 2 [file 12913_2024_10889_MOESM2_ESM.pdf]

Consolidated criteria for reporting qualitative research (COREQ): a 32-item checklist for interviews and focus groups

Domain 1: Research team and reflexivity

Personal Characteristics

1. Interviewer/facilitator. Which author/s conducted the interview or focus group?

In the "Methods" section, under the heading "The interviews" on page 13.

2. Credentials. What were the researcher's credentials? E.g. PhD, MD

In the "Methods" section, under the heading "The interviews" on page 13.

3. Occupation. What was their occupation at the time of the study?

In the "Methods" section, under the heading "The interviews" on page 13.

4. Gender. Was the researcher male or female?

In the "Methods" section, under the heading "The interviews" on page 13.

5. Experience and training. What experience or training did the researcher have?

In the "Methods" section, under the heading "The interviews" on page 13.

Relationship with participants

6. Relationship established. Was a relationship established prior to study commencement?

In the "Methods" section, under the heading "Recruitment and participants" on page 12.

7. Participant knowledge of the interviewer. What did the participants know about the researcher? e.g. personal goals, reasons for doing the research.

In the "Methods" section, under the heading "Ethical approval and consent to participate" on page 15.

8. Interviewer characteristics. What characteristics were reported about the interviewer/facilitator? e.g. Bias, assumptions, reasons and interests in the research topic.

In the "Methods" section, under the heading "The interviews" on page 13.

Domain 2: study design

Theoretical framework

9. Methodological orientation and Theory. What methodological orientation was stated to underpin the study? e.g. grounded theory, discourse analysis, ethnography, phenomenology, content analysis.

The conceptual framework is presented in the Method section under the heading "Conceptual framework" on page 9. The methodology is described in the "Methods" section under the heading "Design" on page 12 and under the heading "Coding and data analysis" on page 15.

Participant selection

10. Sampling. How were participants selected? e.g. purposive, convenience, consecutive, snowball.

In the "Methods" section, under the heading "Recruitment and participants" on page 12.

11. Method of approach. How were participants approached? e.g. face-to-face, telephone, mail, email.

In the "Methods" section, under the heading "Recruitment and participants" on page 12.

12. Sample size. How many participants were in the study?

In the "Methods" section, under the heading "Recruitment and participants" on page 12.

13. Non-participation. How many people refused to participate or dropped out? Reasons?

N/A

Setting

14. Setting of data collection Where was the data collected? e.g. home, clinic, workplace.

In the "Methods" section, under the heading "The interviews" on page 13.

15. Presence of non-participants. Was anyone else present besides the participants and researchers?

In the "Methods" section, under the heading "Observation and informal conversations" on page 13.

16. Description of sample. What are the important characteristics of the sample? e.g. demographic data, date.

In the "Methods" section, under the heading "Data collection" on page 12-14.

Data collection

17. Interview guide. Were questions, prompts, guides provided by the authors? Was it pilot tested?

In the "Methods" section, under the heading "The interviews" on page 13.

18. Repeat interviews. Were repeat interviews carried out? If yes, how many?

N/A

19. Audio/visual recording. Did the research use audio or visual recording to collect the data?

In the "Methods" section under the heading "Coding and data analysis" on page 15.

20. Field notes. Were field notes made during and/or after the interview or focus group?

In the "Methods" section, under the heading "Observation and informal conversations" on page 14.

21. Duration. What was the duration of the interviews or focus group?

In the "Methods" section, under the heading "The interviews" on page 13.

22. Data saturation. Was data saturation discussed?

In the "Methods" section, under the heading "Recruitment and participants" on page 12.

23. Transcripts returned. Were transcripts returned to participants for comment and/or correction?

N/A

Domain 3: analysis and findings

Data analysis

24. Number of data coders. How many data coders coded the data?

In the "Methods" section under the heading "Coding and data analysis" on page 15.

25. Description of the coding tree. Did authors provide a description of the coding tree?

In "Table 1" on page 42-44.

26. Derivation of themes. Were themes identified in advance or derived from the data?

In the "Methods" section under the heading "Coding and data analysis" on page 15.

27. Software. What software, if applicable, was used to manage the data?

In the "Methods" section under the heading "Coding and data analysis" on page 15.

28. Participant checking. Did participants provide feedback on the findings?

N/A

Reporting

29. Quotations presented. Were participant quotations presented to illustrate the themes / findings? Was each quotation identified? e.g. participant number.

Throughout the "Results" section and described in "Table 1" on page 44 and in the "Method" section under the heading "Individual decision letters" on page 14.

30. Data and findings consistent. Was there consistency between the data presented and the findings?

Yes.

31. Clarity of major themes. Were major themes clearly presented in the findings?

Yes.

32. Clarity of minor themes. Is there a description of diverse cases or discussion of minor themes?

Yes.
